# An inactivated poliovirus vaccine using Sabin strains produced on the serum-free PER.C6® cell culture platform is immunogenic and safe in a non-human primate model

**DOI:** 10.1016/j.vaccine.2018.09.068

**Published:** 2018-11-12

**Authors:** Viki Bockstal, Machteld M. Tiemessen, Rogier Achterberg, Carlo Van Wordragen, Ad M. Knaapen, Jan Serroyen, Wilfred E. Marissen, Hanneke Schuitemaker, Roland Zahn

**Affiliations:** a Janssen Vaccines and Prevention BV, Archimedesweg 4-6, 2333CN Leiden, the Netherlands; b Merus N.V., Yalelaan 62, 3584 CM Utrecht, the Netherlands^2^

**Keywords:** Sabin, Inactivated poliovirus vaccine, IPV, sIPV, Salk, Non-human primates, Immunogenicity, Toxicity, Virus neutralizing assay

## Abstract

**Background:**

The World Health Organization recommends the development of affordable next-generation inactivated poliovirus vaccines (IPV) using attenuated poliovirus Sabin strains. Previously, we introduced a novel PER.C6® cell culture platform, which allows for high yield production of an affordable trivalent Sabin IPV vaccine.

**Methods:**

Immunogenicity and safety of this novel PER.C6®-based Sabin-IPV (sIPV) was assessed in rats and non-human primates (NHPs). NHPs received one of four different dose dilutions vaccine according to current human schedule (three prime-immunizations and one boost immunization). For comparison, NHPs received commercially available reference Salk IPV or sIPV.

**Results:**

Dose-dependent immunogenicity and good tolerability was observed for the PER.C6®-based sIPV formulations in rats and NHPs. In NHPs, the lowest tested dose that induced anti-Sabin virus-neutralizing antibody titers that were non-inferior to commercial sIPV after three immunizations was 5-7.5-25 D-antigen units for type 1, 2 and 3 respectively.

**Discussion:**

PER.C6®-based sIPV induced comparable immunogenicity to commercial Salk IPV and sIPV vaccines in NHPs. Together with the absence of any preclinical safety signals, these data warrant further testing in clinical trials. sIPV produced on the PER.C6® cell platform could be one solution to the need for an affordable and immunogenic IPV to achieve and maintain global polio eradication.

## 1 Introduction

As global eradication of poliomyelitis edges closer there is a need for new, affordable vaccines to replace the oral live-attenuated poliovirus vaccine (OPV) based on Sabin strains and conventional inactivated poliovirus vaccines (cIPV) based on Salk strains. Both OPV and cIPV vaccines have been used to control polio until now. However both vaccines have distinct disadvantages for future use [1]. OPV can revert to neurovirulent forms that may cause Vaccine-Associated Paralytic Poliomyelitis (VAPP) in vaccinees or their contacts. In addition, OPV revertants with high transmissibility can circulate in a poorly vaccinated population, causing outbreaks of disease and hence risking the completeness of eradication of poliomyelitis [2], [3]. cIPV by definition cannot revert to neurovirulence, but its use is constrained by the cost of production and insufficient supply for global needs [4], [5]. In addition, because IPV Salk strains are neurovirulent prior to inactivation, they represent a potential biosafety hazard should they escape from a manufacturing facility. Therefore, there is an urgent need for cheaper and safer IPV production processes in order to facilitate polio eradication and to ensure global availability of vaccines towards and in the post-eradication era. To mitigate this risk and facilitate safe vaccine production globally, the World Health Organization (WHO) recommends using the attenuated Sabin poliovirus strains for the development of an affordable next-generation IPV [5].

We previously introduced the PER.C6® cell line as an efficient alternative to the traditional Vero cell culture platform to produce large quantities of Sabin IPV at relatively small footprint [6]. Indeed, we showed a substantially higher (10-fold on average) volumetric productivity of sIPV using the PER.C6® cell platform in terms of infectious titer and D-antigen content compared to the Vero cell platform [6]. The data demonstrated that the PER.C6® cell culture platform is more efficient as cell substrate than the Vero platform, allowing low-cost, high yield production of antigenic Sabin vaccines.

The Wistar rat model is usually the preferred model of choice to test the potency of IPV vaccines. However, there are two challenges in using the Wistar rat model for Sabin strain IPVs. One is that Sabin type 2 poliovirus shows lower immunogenicity compared to the Salk type 2 poliovirus in this rat model [7]. Secondly, there is no international reference standard currently available for testing sIPV, which greatly hinders the interpretation of dose-finding studies using new sIPV vaccines in rats.

Because non-human primates (NHPs) are more closely related to humans than rats, it is likely that the outcome of a dose-finding immunogenicity study that mimics the human IPV immunization schedule of 3 prime- and 1 boost vaccination would be more predictive than other preclinical models. Hence, researchers working with sIPV vaccines generated on the Vero cell platform conducted preclinical dose-finding studies in cynomolgus monkeys before entering clinical trials [8]. An immunogenicity study performed in NHP with a quadrivalent combined diphtheria-tetanus-acellular pertussis-sIPV vaccine (DTaP-sIPV) showed a comparable neutralizing antibody response induced by Sabin types 1, 2 and 3 in DTaP-sIPV to the response induced by DTaP-cIPV and a stand-alone cIPV [9]. In addition, the Institute of Medical Biology in Kunming, China, who brought the first stand-alone sIPV to the market in 2015, published an immunogenicity study in rhesus macaques showing that their DTaP-sIPV candidate vaccine induced neutralizing antibody titers that were similar to those generated by the control DTaP-cIPV and stand-alone sIPV [10]. Immunogenicity in humans was subsequently demonstrated in phase II and III clinical trials [11], [12], [13].

In view of these results, we conducted a dose-finding study of the novel PER.C6®-produced sIPV in a cynomolgus monkey model. After initial dose selection and safety assessment of the novel sIPV in rats, we compared the immunogenicity of four different dose dilutions to commercial cIPV and sIPV reference vaccines in NHP, and determined the lowest sIPV dose that was non-inferior after three priming doses.

## 2 Methods

### 2.1 Vaccines

Sabin virus reference strains were acquired from the National Institute for Biological Standards and Control in the United Kingdom (cat#01/528, 01/530 and 01/532). Mahoney strain was purchased at the European Virus Archive. MEF-1 and Saukett strains were obtained from Crucell Sweden (formerly Swedish national laboratory, SBL). Sabin viruses were produced at 10 L scale in PER.C6® cell culture using an intensified process developed to allow the cultivation at high cell densities [6]. Upon the harvest, cell debris and impurities were removed in clarification filtration steps. Virus was further purified through affinity binding and size exclusion chromatography steps. Inactivation was achieved by incubation at 37 °C in the presence of glycine and formaldehyde. All stocks were sequenced to confirm identity and the presence of attenuating mutations. Formulation of trivalent formulations was performed using M199 medium containing 2-phenoxyethanol. Aliquots of the prepared vaccines were stored at −80 °C for post-hoc control measurement of D-antigen unit (DU) content. The DU content of the sIPV material was determined by an in-house D-antigen enzyme-linked immunosorbant assay (ELISA). Reference vaccines were cIPV (Sanofi Pasteur, Lyon, France) and sIPV (Institute of Medical Biology, Kunming, China).

### 2.2 Animals

Wistar rats (Crl:Wi) were purchased from Charles River Laboratories and housed at the animal facility of Janssen Pharmaceutica, Beerse, Belgium, an AAALAC-accredited facility.

Two to three year-old female cynomolgus monkeys (*Macaca fascicularis*) were used to measure immunogenicity of sIPV. The in-life phase part of this study was performed by Charles River Laboratories (CRL) in Edinburgh, an AAALAC-accredited facility, and the study design was approved by the UK home office and conformed to the regulations of Animal Care and Use as described in the EU Directive 2010/63/EU.

### 2.3 Non-clinical safety testing in Wistar rats

A Good Laboratory Practice (GLP) repeated dose combined toxicity and local tolerance study was conducted in Wistar rats, in line with applicable guidelines [14]. sIPV (17-40-126 DU) was administered intramuscularly once every 2 weeks for 8 weeks (5 injections, 15 animals/sex/group). The vaccine volume of 0.5 mL was administered as 2x0.25 mL injections per dosing day per animal (one injection in each hind leg). Control animals received saline according to the same regimen. Animals were either sacrificed 2 days after the last injection (10 animals/sex/group), or following a 3-week recovery period after the last injection (5 animals/sex/group). Mortality, clinical-, skin- and eye observations, body temperature, body weight, weight gain, food consumption, hematology, coagulation, clinical chemistry, urinalysis, immunogenicity, organ weights, gross pathology and histopathology were examined.

### 2.4 Potency testing in the Wistar rat model

Rats were immunized once with one of four dilutions of PER.C6®-based sIPV or the European reference standard BRP2, a cIPV (European Directorate for the Quality of Medicines and Healthcare). The neat dose of BRP2 was prepared by diluting the concentrated material in a 1:5 dilution to obtain one human dose (40-8-32 DU). Ten animals in each dosage group received a total dose volume of 0.5 mL divided between the hind legs (0.25 mL per leg). Serum was collected 1 to 3 days prior to vaccination and 21 days post-vaccination for measurement of virus neutralizing antibody titers.

### 2.5 Immunogenicity testing in cynomolgus monkeys

Cynomolgus monkeys were immunized four times by intramuscular injection, i.e. mimicking the human vaccine regimen. In total, 36 female animals were used, six animals per experimental group. Per group a specified sIPV dose was administered, with a high dose (20-30-100 DU), mid-dose (10-15-50 DU), mid-low dose (5-7.5-25 DU) or low dose (2.5-3.75-12.5 DU) of the PER.C6®-based sIPV, or one full human dose of commercial cIPV or sIPV. The total injection volume of 0.5 mL was administered into the left or right, lower or upper thigh, depending on the time point of immunization. The three prime immunizations were spaced 3 weeks apart and the boost immunization was provided 11 weeks after the third immunization. Serum was collected prior to every prime immunization and 3 weeks after the final immunization (at week 20 of the study) for measurement of neutralizing antibody titers.

### 2.6 Virus neutralization assay (VNA)

Titers of type-specific poliovirus-neutralizing antibodies in sera were determined by a micro-neutralization test according to the WHO protocol [15] using 100 Tissue culture infectious dose (TCID_50_) of challenge virus (Mahoney, MEF-1 and Saukett or Sabin 1, 2 and 3) on Vero cells. A series of eight 2-fold dilutions of sera were made to which 100 TCID50 of virus was added. For rat sera samples, a start dilution of 1:8 for type 1, 1:64 for type 2 and 1:4 for type 3 was used. For samples from cynomolgus monkeys, a start dilution of 1:4 was used for all types. Virus-serum dilutions were incubated for 3 h at 37 °C prior to addition of 3x10^4^ Vero cells per sample. The samples were incubated at 37 °C for 5 days (Mahoney, MEF-1 and Saukett) or at 32,5 °C for 7 days (Sabin 1, 2 and 3) prior to readout of cytopathic effect by inverted light microscopy. Each serum sample was run in triplicate.

### 2.7 IPV IgG multiplex assay

Anti-poliovirus type 1, 2 and 3-specific total IgG titers were measured in a multiplex assay. Multi-array plates were custom coated by Meso Scale Discovery with mouse monoclonal anti-poliovirus type 1 (6F171), type 2 (24E2) and type 3 (45D5) antibodies (Santa Cruz Biotechnology, Santa Cruz, CA). Multi-array plates were blocked with PBS (GIBCO) containing 1% BSA (Sigma-Aldrich) for 30 min. Plates were washed with PBS containing 0.05% Tween20 (Merck) after which a mix of inactivated Mahoney, MEF-1, and Saukett were diluted in PBS containing 0.005% Tween20 and added to the plates for 1 h. After washing, samples were diluted in PBS containing 0.005% Tween20 1% BSA, and added to the plates. After 2 h of incubation plates were washed and SULFO-TAG labeled goat polyclonal anti-rat IgG antibody (MSD, Gaithersburg, MD) diluted in PBS containing 0.05% Tween20 was added to the plates, which were incubated for 1 h. After washing, Read Buffer T (MSD, Gaithersburg, MD) was added to the plates which were measured in the MESO Sector S 600 (MSD, Gaithersburg, MD). Assays were performed at room temperature.

### 2.8 Statistical analysis

Relative potency in the rat model was calculated based on the number of seroconverting animals for each vaccine in relation to the reference. Seroconversion was defined as the mid-point on a log2 scale of the minimum and maximum geometric mean titers based on a minimum of three repeated tests with the reference vaccine [16]: ≥56, ≥454, ≥47 for Sabin types 1, 2 and 3 respectively. A probit regression model was applied to this binary seroconversion response variable. The model contained vaccine group and log-transformed (ln) dose as explanatory variables. A vaccine is considered to be comparable to the reference if the upper limit of the 95% confidence interval (CI) of the relative potency estimate was >1.0.

In the cynomolgus monkey model, an analysis-of-variance for potentially censored observations (Tobit regression) with group as explanatory variable was performed. The non-inferiority analysis consisted of comparing the lower limit of the 95% CI of the difference in log_10_ titer to a 4-fold non-inferiority margin (i.e., a −0.6 difference in log_10_ titer).

All analyses were performed using R 3.1.1 software [17].

## 3 Results

### 3.1 Assessment of immunogenicity and toxicity of PER.C6®-based sIPV in rats

Monovalent vaccines for Sabin type 1, 2 and 3 were successfully produced on the PER.C6® cell culture platform. The immunogenicity of each monovalent vaccine was tested in a Wistar rat model by determining the neutralizing antibody response 3 weeks after a single immunization. For vaccines using the attenuated Sabin strains it has been shown that a DU/dose of 3/100/100 for types 1, 2 and 3 was similarly antigenic and immunogenic compared to wild polio strain vaccines [18]. Therefore, the selected starting dose for the PER.C6®-based monovalent vaccines was set at 7, 85, 85 DU/dose for type 1, 2 and 3 respectively.

**Fig. 1:**
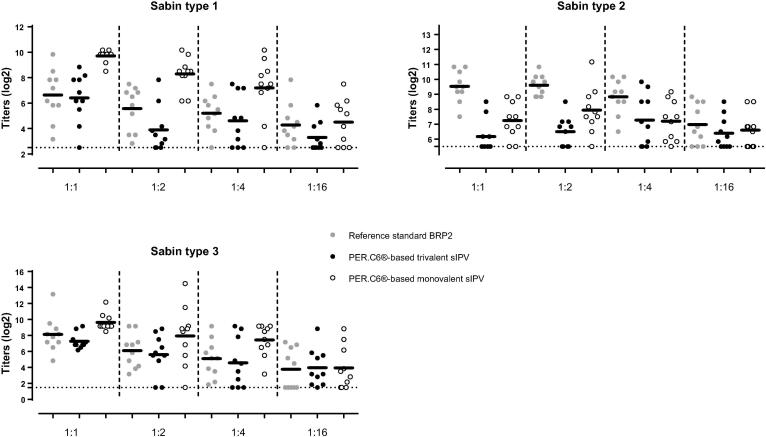
Immunogenicity of monovalent and trivalent PER.C6®-based Sabin IPV (sIPV) for Sabin poliovirus type 1, 2 and 3 compared to the reference standard BRP2 in the Wistar rat model. Sabin virus neutralizing titers were determined three weeks following vaccination of Wistar rats with four serial dilutions of PER.C6®-based monovalent and trivalent sIPV or four serial dilutions of reference standard BRP2. Starting dose for the monovalent sIPV vaccines was 7 DU/dose, 85 DU/dose and 85 DU/dose for type 1, 2 and 3 respectively. The starting dose of the PER.C6®-based trivalent sIPV was 5/62/65 DU/dose). Representative results of one out of two independent experiments are shown in the graphs. Dots show results from individual animals. Dotted lines show the lower limit of quantification. Horizontal lines show geometric means. The relative potency of the monovalent and trivalent PER.C6®-based sIPV product compared to the reference standard BRP2 is indicated in the table below, where a vaccine was considered to be comparable to the reference if the upper limit of the 95% confidence interval (CI) of the relative potency estimate was >1.0.

The monovalent formulations of PER.C6®-based sIPV type 1 and type 3 were found to induce an equally potent neutralizing antibody response compared to the Salk-based BRP2 reference, starting dose of 40/8/32 (Fig. 1). The dosing tested for monovalent Sabin type 2 did not result in comparable immunogenicity to BRP2. A dose-response relationship was observed for Sabin types 1 and 3, but was absent for Sabin type 2 after sIPV vaccination.

Based on the outcome of the monovalent sIPV testing, that showed a high immunogenicity for type 1 and 3 compared to BRP2 and no clear dose response of type 2, the DU was lowered in the trivalent product to a starting dose of 5/62/65 DU/dose for type 1, 2 and 3 respectively. The trivalent sIPV vaccine induced comparable immunogenicity for Sabin type 1 and type 3 to the BRP2 reference. The potency of type 2 in the trivalent mixture remained lower than BRP2 at a level comparable to monovalent sIPV type 2.

Toxicity and local tolerance of PER.C6®-based trivalent sIPV was assessed by immunizing rats with a high dose of PER.C6®-based sIPV (17-40-126 DU) at five consecutive time points over a period of 8 weeks (i.e. animals were vaccinated every 2 weeks). High anti-poliovirus type 1, 2 and 3 IgG ELISA titers were observed in all animals (Supplementary Fig. 1). No test article-related mortality was noted, and there were no relevant local (including skin evaluation at site of injection using Draize scoring) and systemic clinical observations, as well as eye observations. In addition, there were no relevant vaccine related changes in body temperature, body weight, body weight gain, food consumption, clinical chemistry, coagulation or urinalysis.

A minimal increase in the number of neutrophils and white blood cells was noted in male animals. Except for enlarged draining medial iliac lymph nodes in few animals, there were no other relevant gross necropsy findings. Histopathologically, the draining lymph nodes had increased germinal center development and increased medullary cellularity, sometimes associated with an increased weight of the (medial iliac) lymph node, indicating an immunologic response to vaccination. An increased incidence of inflammatory cell infiltrates was noted in the intramuscular injections sites. The severity of these injection site findings was considered (very) low in all cases. At the end of the 3-week treatment free period following the last injection, lower incidences of the inflammatory changes at the injection sites where noted. Also the changes noted in the draining lymph node showed lower incidences and/or severity at the end of the recovery period. Overall, PER.C6®-based sIPV was well tolerated by the animals. The vaccine related effects showed (ongoing) recovery following a 3 week treatment free period after the last injection.

### 3.2 Dose selection of PER.C6®-based sIPV in rats

To select the optimal dose range of trivalent PER.C6®-based sIPV for a subsequent NHP study, a rat potency study was performed with four serial dilutions of sIPV (starting dose 10-15-50 DU). The trivalent formulation of 10-15-50 DU/dose was selected to explore the possibility of lowering the dose of types 2 and 3 while ensuring high type 1 immunity, which would be beneficial from a cost of goods perspective.

**Fig. 2:**
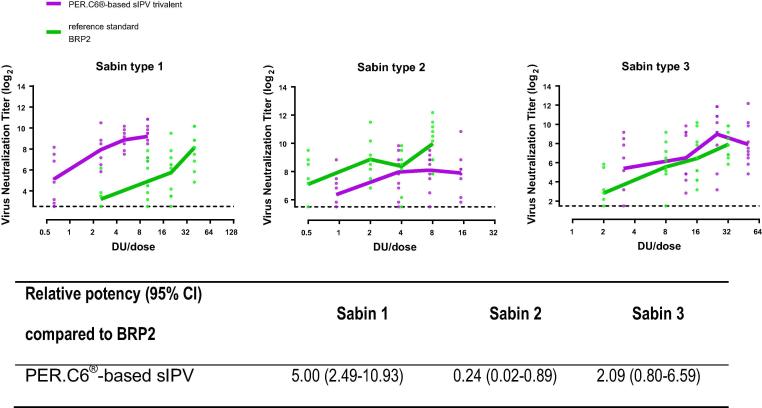
Immunogenicity and relative potency of a trivalent PER.C6®-based Sabin IPV (sIPV) as measured by Sabin neutralizing antibody titers relative to the reference standard BRP2 in the Wistar rat model. Wistar rats were intramuscularly immunized with four serial dilutions of PER.C6®-based sIPV (starting dose 10-15-50 DU/dose) or reference standard BRP2 (starting dose 40-8-32 DU/dose). Three weeks after immunization, sera were collected and analyzed with a Sabin neutralizing antibody assay. Virus neutralizing antibody titers are shown for each animal at each dilution (dots represent individual animals) and the dotted line represents the assay start dilution. The relative potency of the trivalent PER.C6®-based sIPV product compared to the reference standard BRP2 is indicated in the table below, where a vaccine was considered to be comparable to the reference if the upper limit of the 95% confidence interval (CI) of the relative potency estimate was >1.0.

Immunogenicity and a dose titration effect could be demonstrated for all three polio virus types in the trivalent PER.C6®-based sIPV. A comparison to the reference standard BRP2 showed that the trivalent PER.C6®-based sIPV formulation was more, less or equally immunogenic to BRP2 for Sabin type 1, type 2 and type 3 respectively (Fig. 2). To identify an alternative immunological readout for single poliovirus type-specific VNA to be used in rat potency studies, we developed a multiplex binding antibody assay in parallel for several studies. The specific binding IgG antibody titers to Salk poliovirus measured by the multiplex binding antibody assay showed a good correlation to the Sabin VNA, correlation factors were 0.85; 0.88 and 0.89 for type 1, 2 and 3 respectively (Supplementary Fig. 2). This indicates that a multiplex binding antibody assay is likely to serve as a rapid alternative for single, poliovirus type-specific VNA.

### 3.3 PER.C6®-based sIPV dose-finding in cynomolgus monkeys

**Fig. 3:**
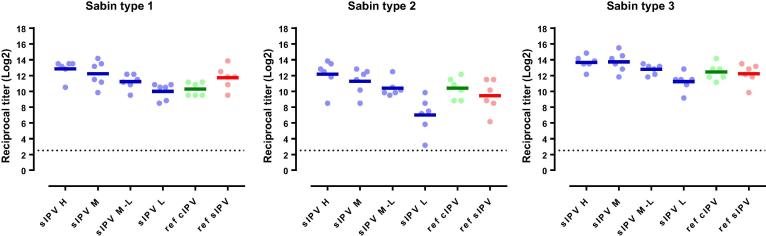
Sabin virus neutralizing antibody titers against poliovirus type 1, 2 and 3, three weeks after the third immunization in cynomolgus monkeys immunized with different polio vaccines. Cynomolgus monkeys (6 per group) were intramuscularly immunized with PER.C6®-based Sabin IPV (sIPV), or a reference Salk IPV (ref cIPV, *Imovax*, Sanofi) or a reference sIPV (ref sIPV, Kunming). For both commercial vaccines, one full human dose was used, and for the PER.C6®-based sIPV four different D-antigen unit dose formulations were tested, annotated as H, M, M-L and L. The different dose formulations represent: H = high dose formulation (20-30-100 DU), M = mid dose (10-15-50 DU), M-L = mid low dose (5-7.5-25 DU), L- low dose (2.5-3.75-12.5 DU). Three weeks after the third immunization sera were collected and analyzed with Sabin virus neutralizing antibody assays. The titration endpoint per animal is presented as a reciprocal of the titer. Dots represent individual animals. Horizontal lines show geometric means. The dotted line is the cut-off for seroprotection in humans (serum dilution of 1:8).

To determine the immunogenicity and dose of PER.C6®-based sIPV in cynomolgus monkeys that are more predictive for the immune response in humans, four different dose formulations were administered in an immunization regimen similar to the human primary immunization regimen. The regimen consisted of three prime immunizations, 3 weeks apart, and one late boost immunization 11 weeks post-dose 3. Immunizations were performed with four different dose formulations starting at a high dose (20-30-100 DU) and subsequent two-fold dilutions (high, mid, mid-low and low, as explained in the legend of Fig. 3) of the PER.C6®-based sIPV, or groups dosed with commercial cIPV or commercial sIPV as reference vaccines at full human dose.

**Fig. 4:**
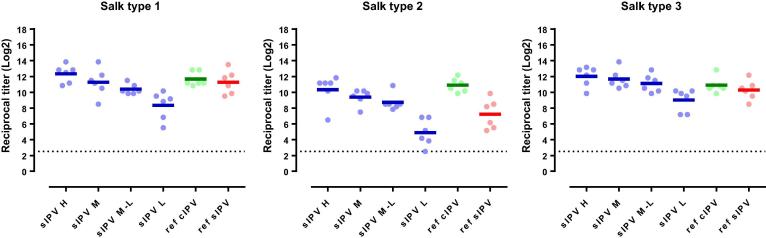
Non-inferiority of PER.C6®-based sIPV compared to Sabin IPV (sIPV) and Salk IPV (cIPV) commercial reference vaccines based on Sabin virus neutralizing antibody titers after three immunizations (at week 9). Sera from cynomolgus monkeys, immunized 3 times with one of the four dose formulations of PER.C6®-based sIPV or commercial cIPV or sIPV, were analyzed with Sabin viral neutralizing assay to determine Sabin virus neutralizing antibody titers. The four different D-antigen unit dose formulations were high dose formulation (20-30-100 DU), mid dose (10-15-50 DU), mid low dose (5-7.5-25 DU) and low dose (2.5-3.75-12.5 DU). To assess non-inferiority between the different vaccines, an area under the curve analysis at week 9 was performed. A vaccine formulation is regarded non-inferior compared to the reference vaccine if the lower limit of the 95% confidence interval (CI) of the difference in log_10_ titer is above −0.6 difference in log_10_ titer (indicated by the vertical dotted line).

**Fig. 5:**
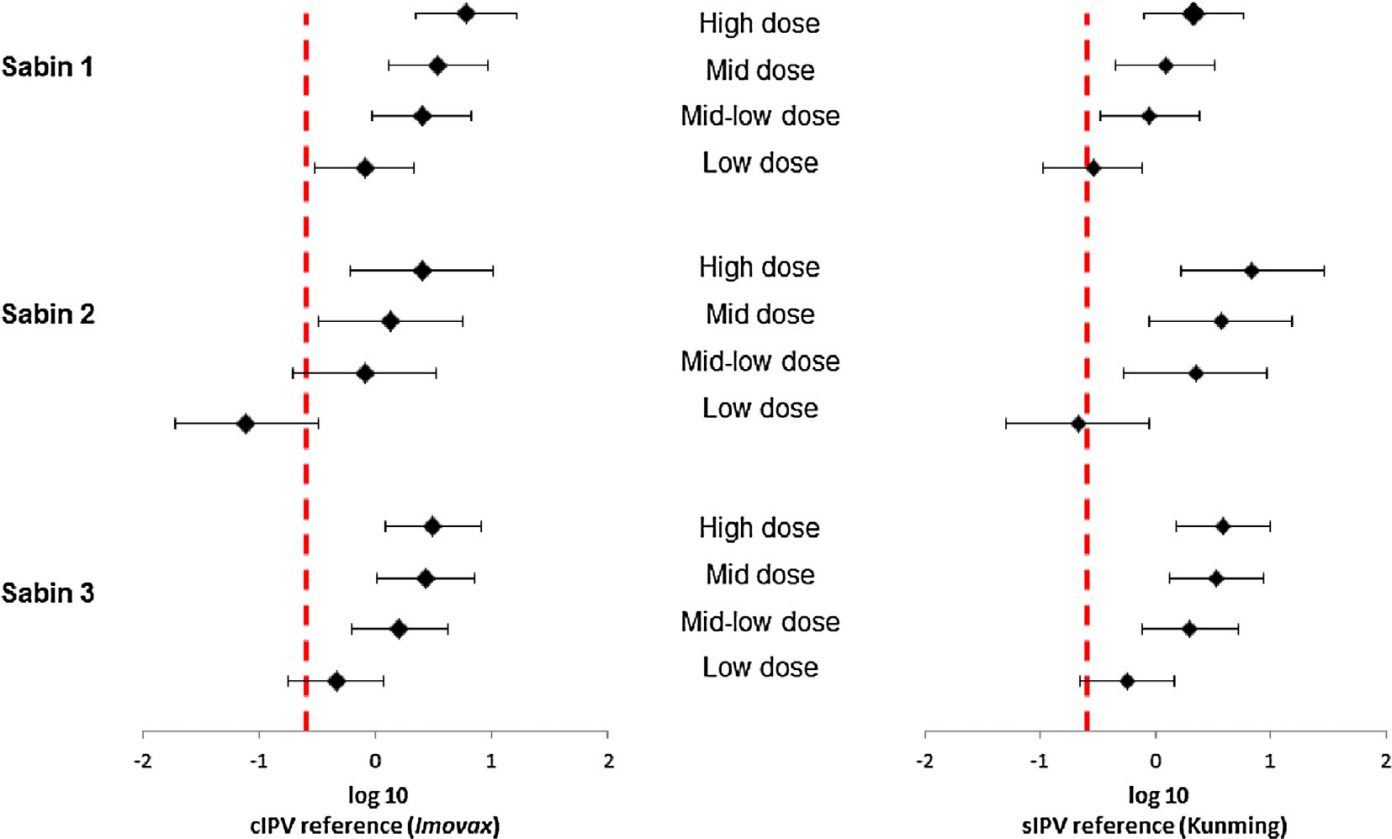
Salk virus neutralizing antibody titers against poliovirus type 1, 2 and 3, three weeks after the third immunization in cynomolgus monkeys immunized with different polio vaccines. Cynomolgus monkeys (6 per group) were intramuscularly immunized with PER.C6®-based sIPV, or a reference Salk IPV (ref cIPV, *Imovax*, Sanofi) or a reference Sabin IPV (ref sIPV, Kunming). For both commercial vaccines, one full human dose was used, and for the PER.C6®-based sIPV four different D-antigen unit dose formulations were tested, annotated as H, M, M-L and L. The different dose formulations represent: H = high dose formulation (20-30-100 DU), M = mid dose (10-15-50 DU), M-L = mid low dose (5-7.5-25 DU), L-low dose (2.5-3.75-12.5 DU). Three weeks after the third immunization sera were collected and analyzed with Salk virus neutralizing antibody assays. The titration endpoint per animal is presented as a reciprocal of the titer. Dots represent individual animals. Horizontal lines show geometric means. The dotted line is the cut-off for seroprotection in humans (serum dilution of 1:8).

After the three primary series immunizations (21 days after the 3rd immunization), a dose-response relationship was observed for the PER.C6®-based sIPV-induced antibody responses for all three poliovirus types for both Sabin and Salk (Fig. 3, Fig. 5). The lowest trivalent sIPV dose that induced anti-Sabin VNA titers non-inferior to the sIPV reference vaccine post-dose 3, was 5-7.5-25 DU (mid-low dose) (Fig. 4, right panel). For this mid-low sIPV dose, non-inferiority was also demonstrated compared to the cIPV reference vaccine for Sabin types 1 and 3, but not for Sabin type 2 (Fig. 4, left panel). For Sabin type 2 non-inferiority to the cIPV reference vaccine could be demonstrated using the mid dose (10-15-50 DU). Next to the primary VNA readout using homologous Sabin strains as assay virus we also investigated the response using Salk input virus. Here we confirmed the induction of a dose response and the results of the Sabin virus comparability to the reference vaccines sIPV and cIPV (Fig. 5). All dose formulations induced high anti-Salk VNA titers that were well above the limit of protection in humans (2.5log_2_, neutralization at serum dilution of 1:8 in VNA, indicated by the dotted line in Fig. 5).

**Fig. 6:**
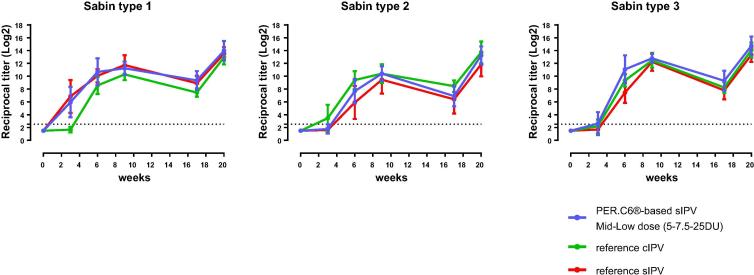
Kinetics of the immune response for the mid-low dose of PER.C6®-based Sabin IPV (sIPV) and commercial reference Salk IPV (cIPV) and sIPV up to week 20 after vaccination. Cynomolgus monkeys were immunized at week 0, 3, 6 and 17 with the indicated vaccines. Sera were collected and virus neutralizing antibody titers for Sabin were measured prior to each immunization and at week 9 and 20 after start of the study. The mean (+/− SD) titration endpoint per group (n = 6) is presented as a reciprocal of the titer. The dotted line is the cut-off for seroprotection in humans (serum dilution of 1:8). Footnote: reference cIPV = *Imovax*, Sanofi; reference sIPV = commercial sIPV, Kunming.

An approximate 2 log_2_ decrease in VNA titers was observed in sera of all groups for both Sabin and Salk and for all three poliovirus types, before the boost immunization at week 17 (Supplementary Tables 1 and 2). This is in line with typical rates of waning immunity in human sera of approximately 1 log_2_ per month [19]. A strong boost effect was observed after the 4th immunization for all four sIPV formulations (Supplementary Tables 1 and 2 and Supplementary Fig. 3). Kinetics of the immune response for the 5-7.5-25 DU (mid-low dose) show high VNA titers after each dose that were within the range induced by the cIPV and sIPV reference vaccines in both VNA assays performed (Fig. 6 (Sabin VNA) and 7 (Salk VNA). Exploratory non-inferiority analyses suggested comparable responses up to week 20 between the high and mid sIPV doses and the cIPV reference vaccine for all three polio types, and for the high, mid and mid-low doses compared to the sIPV reference vaccine (Supplementary Fig. 4).

**Fig. 7:**
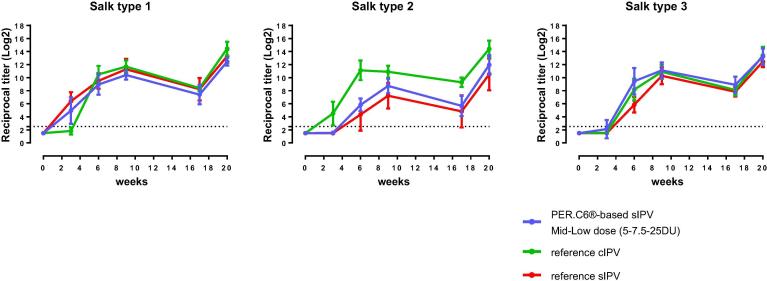
Kinetics of the immune response for the mid-low dose of PER.C6®-based Sabin IPV (sIPV) and commercial reference Salk IPV (cIPV) and sIPV up to week 20 after vaccination. Cynomolgus monkeys were immunized at week 0, 3, 6 and 17 with the indicated vaccines. Sera were collected and virus neutralizing antibody titers for Salk were measured prior to each immunization and at week 9 and 20 after start of the study. The mean (+/− SD) titration endpoint per group (n = 6) is presented as a reciprocal of the titer. The dotted line is the cut-off for seroprotection in humans (serum dilution of 1:8). Footnote: reference cIPV = *Imovax*, Sanofi; reference sIPV = commercial sIPV, Kunming.

There was no adverse effect of vaccine treatment apparent in any animal at any time during the course of the study in terms of clinical observations, dermal scoring of the injection sites, body weight and body temperature. Overall, PER.C6®-based sIPV was well tolerated and immunogenic in the NHP model.

## 4 Discussion

With the progression of the Global Poliomyelitis Eradication Initiative (GPEI), sufficient supplies of a safe, immunogenic and affordable IPV, based on Sabin strains, becomes more urgent. Recently, we have identified the PER.C6® cell line, as a suitable platform for production of sIPV in large quantities [6]. As a precursor to clinical assessment of this novel PER.C6®-based IPV in humans, we have focused on preclinical models studying sIPV-specific systemic humoral immunity.

We have not investigated the ability of the vaccine to induce mucosal immunity in animals given the complexity of the human immune responses to IPV and OPV vaccination. This complexity is further increased by the current global vaccination situation that is providing adequate protection insome children receiving schedules with IPV alone, some only bivalent OPV and some mixed schedules of both IPV and bOPV [20], [21]. GPEI policy currently indicates that IPV is expected to be supportive of OPV induced immunity in the current intra-eradication period. Once polio is eradicated, IPV induced immunity is expected to be sufficient for protection of vaccinees [22], [23] hence our focus on the systemic immune response should suffice to assess immunogenicity and safety of a novel sIPV vaccine.

As a first safety and immunogenicity test for trivalent PER.C6®-based sIPV we explored immunogenicity in different dose formulations in a Wistar rat model and a cynomolgus macaque model. As we only used mature animals we do not address the effect of IPV maternal antibodies on PER.C6®-based sIPV immunogenicity, which may hamper sIPV vaccine take similar to current conventional IPV take in infants up to 14 weeks of age. NHP models closely mimic the human immune response, which enables the evaluation of human vaccination regimens, and are therefore more likely to be representative of the immunogenicity of sIPV in humans [8], [9], [24].

Potency studies for Salk IPV have always been performed in the rat model. However, previous investigations have shown that the Wistar rat model is not optimal for dose-finding studies of new sIPV vaccines, especially for type 2. Indeed, different immunogenicity profiles for poliovirus type 2 between Salk and Sabin based vaccine strains were observed in this rat model, while these vaccines showed comparable immunogenicity in Phase II and III clinical trials [7], [11], [12], [25], [26]. Consistent with these observations, we also observed lower immunogenicity and reduced potency of Sabin type 2 versus BRP2 in the Wistar rat model (Fig. 1, Fig. 2). However, although lower immunogenicity was demonstrated in the rat model, the immunogenicity of the PER.C6®-based sIPV type 2 responses in the NHP were immunogenic and equally immunogenic compared to both Salk- and Sabin-based reference vaccines (Fig. 3, Fig. 5). Therefore, an appropriate potency assessment and dose-finding of a novel trivalent sIPV vaccine appears to require an NHP model as the B cell repertoire in rats appear to be different. Rat potency studies may suffice for release testing and primary quality assessment of novel IPV products, but can lead to an underestimation of the response to a specific or multiple polio type. Other animal models next to NHP models may be also suitable for potency estimation, but have not been explored by us.

A full GLP toxicity study in rats showed that PER.C6®-based sIPV was well tolerated, with only mild reactions observed in the injection sites and draining lymph nodes. These responses showed ongoing recovery 3 weeks after the last injection and were considered to reflect a normal, non-adverse response to the vaccine administration. Similarly, in the NHP immunogenicity study, also no safety signals were reported during or after immunization of the animals.

The novel PER.C6®-based sIPV was tested in four dose formulations in NHPs using the human schedule of 3 prime doses and one booster dose. In the Sabin VNA, the mid-low dose of 5-7.5-25 DU was non-inferior to the reference sIPV after three immunizations. The mid-low dose was also non-inferior to the reference cIPV*,* but only for poliovirus types 1 and 3. However, for poliovirus type 2 the geometric means after three immunizations were equal for the mid-low dose PER.C6®-based sIPV and the reference cIPV: 1341 (range 724–5793) for sIPV and 1341 (range 453–4598) for cIPV. Therefore, it is likely that non-inferiority could not be shown for type 2 due to the higher than expected variability in the titers, rather than a true difference in immunogenicity.

With respect to the dose of PER.C6®-based sIPV needed to confer non-inferior immunogenicity (compared to a commercial sIPV) in NHP, the DU per dose of 5/7.5/25 for the three serotypes respectively, was exceptionally low. For other licensed sIPV products good immunogenicity data were reported in phase II and III clinical trials with antigen levels of 15/32/45 (Liao JID 2012) or 15/50/50 (Okada JID 2013) DU/dose. Whilst it is difficult to directly compare antigenicity of the various products due to differences in D-antigen test methods, the results of the PER.C6®-based sIPV suggest that this novel production process yields highly antigenic sIPV. Future studies will be directed towards the combination of the PER.C6®-based sIPV with other routine childhood vaccines in order to achieve a hexavalent vaccine formulation to provide protection at early age. For another sIPV product, the combination with diphtheria toxoid, tetanus toxoid, and an acellular pertussis antigens, was tested in an NHP model and found not to hamper the immunogenicity of the sIPV of the other vaccines [8]. However, this does not exclude immune interference for novel vaccines and novel combinations to achieve a hexavalent vaccine, which therefore should be tested in an appropriate animal model prior to human testing.

Neutralizing IPV antibody titers were measured in both Sabin as well as in wild type (Salk) VNAs. Antibody titers as measured in the VNA against the strain used for immunization (Sabin) are as expected generally slightly higher compared to the non-matching wild type strain (Salk). Despite this small difference, there was a strong correlation in antibody titers as measured by Sabin and Salk VNA, indicating that a Sabin VNA could potentially replace Salk VNA in the future, thereby slightly lifting the pressure of the need of higher containment when using Salk strain based quality testing.

A good correlation between VNA titers and IgG concentrations as measured by the multiplex binding antibody assay was also shown. Compared to VNA, the multiplex assay is simple to perform, less labor-intensive and provides results within 1.5 days compared to 3 times 7 days for VNA. Multiplex is therefore a feasible alternative to Sabin VNA in rat potency release testing once a sufficiently big data set in both assays for release has been established.

In conclusion, a novel PER.C6®-based sIPV induced comparable immunogenicity to commercial cIPV and sIPV vaccines in NHP at a dose of 5-7.5-25 DU. These promising data, together with the absence of any relevant safety signals in the preclinical studies in rats and NHPs, support further investigation of the PER.C6®-based sIPV in clinical trials. sIPV produced on the PER.C6® cell platform could be one solution to the need for an affordable and immunogenic IPV that can be used to achieve and maintain global polio eradication.

## Conflict of interest statement

All authors were employees of Janssen Vaccines and Prevention BV at the time of the study.

## Funding

Costs associated with development of this manuscript were funded by Janssen and the Bill and Melinda Gates Foundation (BMGF).

## Previous presentation of data

The results presented in this manuscript have not been presented previously.
