# Supplementary material for: An inactivated poliovirus vaccine using Sabin strains produced on the serum-free PER.C6® cell culture platform is immunogenic and safe in a non-human primate model
Source: Vaccine. 2018 Nov 12;36(46):6979–87. doi: 10.1016/j.vaccine.2018.09.068 (PMC6219454; doi:10.1016/j.vaccine.2018.09.068)
Supplement: Supplementary data 1 [file mmc1.docx]

**Supplement**

**Supplementary Figure 1:** Mean IgG antibody titers (Multiplex binding assay) after 5 injections with high-dose PER.C6^®^-based Sabin IPV (sIPV) in Wistar rats.

The PER.C6^®^-based sIPV was administered once every 2 weeks by the intramuscular route to Wistar rats, for a period of 8 weeks in total (5 injections). An immunogenicity analysis based on a rat-specific multiplex antibody binding assay measuring anti-poliovirus IgG antibodies specific for poliovirus type 1,2 or 3 was performed on day 58 (i.e. two days after the 5^th^ injection, n=15) and 3 weeks after the last injection (day 77, n=5). PER.C6^®^-based Sabin sIPV was dosed at 17 DU for poliovirus type 1; 40 DU for type 2 and 126 DU for type 3, control animals received formulation buffer. For the analysis of the serum samples, relative potency (RP) in comparison to the standard serum was calculated using a 4PL model. The log10 RP was reported as the titer. R software was used for analysis of the curves of each sample and calculation of the titer. The dotted line represents the lower limit of qualification and the dots show results from individual animals. Horizontal bars indicate the mean value per group.

**Supplementary Figure 2:** Sabin anti-poliovirus types 1, 2 and 3-specific total IgG binding titers measured with multiplex antibody binding assay and correlation with viral neutralizing assay.

Upper panel: Poliovirus type 1-, 2- and 3-specific antibody titers were determined by multiplex antibody binding assay at day 21 post immunization. Lower panel: Comparison of Salk multiplex antibody binding assay versus Sabin VNA for the trivalent PER.C6^®^-based Sabin sIPV product. Tested in three independent experiments. Spearman r was calculated to give a correlation indication. Footnote: Dots represent individual animals. Dotted line is the assay start dilution. DU = D antigen units

**Supplementary Figure 3:** Sabin and Salk virus neutralizing antibody titers against poliovirus type 1, 2 and 3, three weeks after the boost immunization in cynomolgus monkeys immunized with different polio vaccines.

Cynomolgus monkeys (6 per group) were intramuscularly immunized with PER.C6^®^-based sIPV, or a reference Salk IPV (ref cIPV, Imovax, Sanofi) or a reference Sabin IPV (ref sIPV, Kunming). For both commercial vaccines, one full human dose was used, and for the PER.C6^®^-based sIPV four different D-antigen unit dose formulations were tested, annotated as H, M, M-L and L. The different dose formulations represent: H = high dose formulation (20-30-100 DU), M= mid dose (10-15-50 DU), M-L = mid low dose (5-7.5-25 DU), L- low dose (2.5-3.75-12.5 DU). Three weeks after the fourth immunization sera were collected and analyzed with Salk and Sabin virus neutralizing antibody assays. The titration endpoint per animal is presented as a reciprocal of the titer. Dots represent individual animals. Horizontal lines show geometric means. The dotted line is the cut-off for seroprotection in humans (serum dilution of 1:8).

**Supplementary Figure 4:** Non-inferiority of PER.C6^®^-based sIPV compared to Sabin IPV (sIPV) and Salk IPV (cIPV) commercial reference vaccines based on Sabin virus neutralizing antibody titers after the booster immunization.

Cynomolgus monkeys were immunized 4 times (3 prime and 1 booster immunization) with one of the four dose formulations of PER.C6^®^-based sIPV or commercial cIPV or sIPV. The four different D-antigen unit dose formulations were annotated as H, M, M-L and L. The different dose formulations represent: H = high dose formulation (20-30-100 DU), M= mid dose (10-15-50 DU), M-L = mid low dose (5-7.5-25 DU), L- low dose (2.5-3.75-12.5 DU). Sera were collected 3 weeks after the booster immunization and analyzed with Sabin viral neutralizing assay to determine Sabin virus neutralizing antibody titers.

To assess non-inferiority between the different vaccines an area under the curve analysis up to week 20 was performed. A vaccine formulation is regarded non-inferior compared to the reference vaccine if the lower limit of the 95% CI of the difference in log_10_ titer is above -0.6 difference in log_10_ titer (indicated by the vertical dotted line).


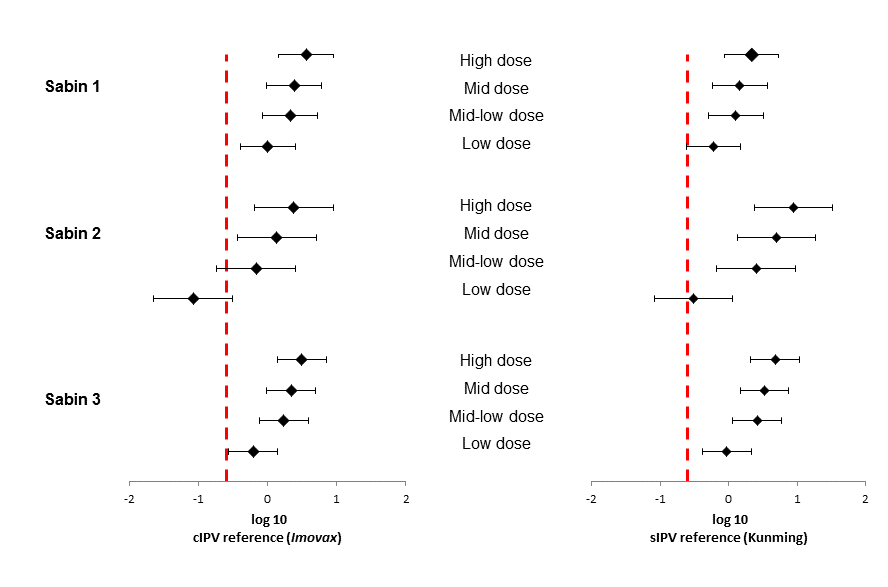


**Supplementary table 1**: Sabin Virus neutralizing antibody titers (VNT) at all time points in sera of cynomolgus monkeys vaccinated with one of four dose formulations of PER.C6^®^-based Sabin IPV (sIPV), or reference Salk IPV (cIPV) or reference sIPV

| **DU/dose**^1^ | **Virus neutralizing antibody titer geometric mean (range)** | | | | | |
| --- | --- | --- | --- | --- | --- | --- |
|  | **Weeks post prime immunization** | | | | | |
|  | **0** | **3** | **6** | **9** | **17 (boost)** | **20** |
| **Sabin 1** | | | | | | |
| **20**-30-100 | LoD | 114  (36-456) | 2389  (912-7298) | 7298  (1448-11585) | 948  (228-3649) | 22295  (9195-46341) |
| **10-**15-50 | LoD | 87  (4-912) | 1064  (228-3649) | 4778  (912-18390) | 553  (91-3649) | 17696  (4598-46341) |
| **5**-7.5-25 | LoD | 61  (LoD-228) | 1625  (114-5793) | 2389  (724-4598) | 645  (228-2299) | 15765  (7298-92682) |
| **2.5**-3.75-12.5 | LoD | 8  (LoD-57) | 276  (23-1149) | 1024  (362-1825) | 147  (91-456) | 8192  (1825-23171) |
| cIPV ref | LoD | 3  (LoD-6) | 376  (91-912) | 1241  (724-2299) | 174  (91-287) | 7883  (3649-23171) |
| sIPV ref | LoD | 114  (14-1149) | 1064  (575-3649) | 3379  (724-14597) | 474  (181-1448) | 11585  (5793-36781) |
| **Sabin 2** | | | | | | |
| 20-**30**-100 | LoD | 9  (LoD-91) | 724  (72-2896) | 4598  (362-14597) | 782  (144-2299) | 31530  (9195-92682) |
| 10-**15**-50 | LoD | 6  (LoD-18) | 362  (14-1825) | 2483  (362-7298) | 219  (23-1448) | 19863  (4598-58386) |
| 5-**7.5**-25 | LoD | 3  (LoD-9) | 203  (72-1825) | 1341  (724-5793) | 119  (23-575) | 9556  (3646-29193) |
| 2.5-**3.75**-12.5 | LoD | LoD | 11  (LoD-724) | 128  (9-912) | 14  (LoD-72) | 1064  (114-9195) |
| cIPV ref | LoD | 11  (LoD-72) | 670  (181-2299) | 1341  (456-4598) | 348  (228-912) | 13515  (1825-46341) |
| sIPV ref | LoD | 3  (LoD-5) | 59  (6-362) | 697  (72-2896) | 84  (18-575) | 3649  (912-18390) |
| **Sabin 3** | | | | | | |
| 20-30-**100** | LoD | 35  (LoD-362) | 5793  (1825-18390) | 13004  (4598-29193) | 1341  (362-4598) | 46341  (18390-116772) |
| 10-15-**50** | LoD | 22  (LoD-91) | 3010  (362-11585) | 13514  (3649-46341) | 948  (287-1825) | 30339  (11585-92682) |
| 5-7.5-**25** | LoD | 6  (LoD-57) | 2128  (181-14597) | 7023  (3649-11585) | 621  (181-2299) | 27029  (5793-92682) |
| 2.5-3.75-**12.5** | LoD | 8  (LoD-114) | 181  (LoD-4598) | 2389  (575-7298) | 174  (36-575) | 9931  (1149-29193) |
| cIPV ref | LoD | 4  (LoD-18) | 645  (362-1149) | 5574  (2299-18390) | 299  (181-724) | 17027  (7298-58386) |
| sIPV ref | LoD | 3  (LoD-4) | 174  (36-456) | 4778  (912-11585) | 220  (45-724) | 10321  (3649-36781) |

^1^ The 3 sIPV doses refer to Type 1, 2 and 3 strains, respectively.

LoD = limit of detection, DU = D-antigen units, cIPV ref = *Imovax* (Sanofi), sIPV ref = Kunming sIPV

**Supplementary table 2**: Salk Virus neutralizing antibody titers (VNT) at all time points in sera of cynomolgus monkeys vaccinated with one of four dose formulations of PER.C6^®^-based Sabin IPV (sIPV), or reference Salk IPV (cIPV) or reference sIPV

| **DU/dose**^1^ | **Virus neutralizing antibody titer (VNT): geometric mean (range)** | | | | | |
| --- | --- | --- | --- | --- | --- | --- |
|  | **Weeks post prime immunization** | | | | | |
|  | **0** | **3** | **6** | **9** | **17 (boost)** | **20** |
| **Mahoney** | | | | | | |
| **20**-30-100 | LoD | 67  (29-228) | 1393  (362-4598) | 5161  (1825-14597) | 474  (114-2299) | 16384  (5793-58386) |
| **10-**15-50 | LoD | 39  (4-228) | 621  (91-229) | 2483  (362-14597) | 196  (36-575) | 11148  (1825-46341) |
| **5**-7.5-25 | LoD | 31  (LoD-114) | 493  (144-2299) | 1341  (912-2896) | 168  (45-724) | 5574  (2896-9195) |
| **2.5**-3.75-12.5 | LoD | 6  (LoD-36) | 161  (9-724) | 323  (45-1149) | 59  (18-114) | 2787  (912-11585) |
| cIPV ref | LoD | 4  (LoD-7) | 1448  (456-4598) | 3251  (1825-7298) | 335  (228-456) | 22295  (9195-46341) |
| sIPV ref | LoD | 84  (29-287) | 724  (228-2299) | 2483  (724-11585) | 299  (91-1825) | 9931  (7298-36781) |
| **MEF-1** | | | | | | |
| 20-**30**-100 | LoD | 8  (LoD-144) | 287  (36-724) | 1290  (91-3649) | 335  (57-1149) | 12040  (724-58386) |
| 10-**15**-50 | LoD | 3  (LoD-7) | 139  (6-2299) | 670  (181-1149) | 128  (14-456) | 12513  (4598-46341) |
| 5-**7.5**-25 | LoD | 3  (LoD-4) | 57  (29-144) | 422  (228-1825) | 51  (9-181) | 3941  (912-18390) |
| 2.5-**3.75**-12.5 | LoD | LoD | 6  (LoD-114) | 26  (LoD-114) | 6  (LoD-36) | 174  (23-1149) |
| cIPV ref | LoD | 23  (5-72) | 2212  (456-9195) | 1896  (912-4598) | 621  (362-1149) | 21453  (11585-92682) |
| sIPV ref | LoD | LoD | 20  (LoD-228) | 149  (36-912) | 28  (LoD-181) | 1448  (287-7298) |
| **Saukett** | | | | | | |
| 20-30-**100** | LoD | 15  (LoD-287) | 1064  (228-5793) | 4096  (912-9195) | 645  (181-1825) | 8848  (3649-18390) |
| 10-15-**50** | LoD | 9  (LoD-45) | 782  (144-2896) | 3251  (1448-14597) | 621  (144-1448) | 8192  (3649-14597) |
| 5-7.5-**25** | LoD | 4  (LoD-29) | 697  (72-3649) | 2212  (912-7298) | 474  (181-1149) | 9556  (2299-23171) |
| 2.5-3.75-**12.5** | LoD | 3  (LoD-7) | 69  (LoD-2299) | 512  (144-1149) | 119  (23-912) | 4598  (575-14597) |
| cIPV ref | LoD | 3  (LoD-4) | 287  (91-1149) | 1896  (912-7298) | 277  (114-456) | 10726  (2896-29193) |
| sIPV ref | LoD | LoD | 57  (18-181) | 1241  (362-4598) | 228  (114-456) | 5574  (2299-9195) |

^1^ The 3 sIPV doses refer to Type 1, 2 and 3 strains, respectively.

LoD = limit of detection, DU = D-antigen units, cIPV ref = *Imovax* (Sanofi), sIPV ref = Kunming sIPV
